# On Removal of the Stomach

**Published:** 1899-04-08

**Authors:** 


### ON REMOVAL OF THE STOMACH.

In the *Medical Record* of New York of March 11th there appears a full account of Carl Schlatter's well-known case of complete removal of the stomach, carrying the history up to the death of the patient, which took place last October. The woman lived for nearly fourteen months after the operation without a vestige of a stomach, finally succumbing to general cancerous infection proceeding from a carcinoma of the mesenteric lymph glands. She had continued to gain in weight and strength until the cancerous cachexia sapped the growing vitality, and thus her death appeared to be connected neither with the surgical interference nor with the total abolition of stomachic digestion. As Dr. Schlatter says, "for an entire year had the fifty-seven-year-old woman lived free from suffering, without a stomach, and had even gained notably in body weight in the time. Up to within the last ten weeks of her life she had been able to go about outside the hospital, but then with the appearance of cachectic symptoms had rapidly succumbed to her malady."

